# Insights into molecular structure, genome evolution and phylogenetic implication through mitochondrial genome sequence of *Gleditsia sinensis*

**DOI:** 10.1038/s41598-021-93480-6

**Published:** 2021-07-21

**Authors:** Hongxia Yang, Wenhui Li, Xiaolei Yu, Xiaoying Zhang, Zhongyi Zhang, Yuxia Liu, Wenxiu Wang, Xiaoxuan Tian

**Affiliations:** 1 State Key Laboratory of Component-Based Chinese Medicine, Tianjin University of Traditional Chinese Medicine, Tianjin, 300193 China; 2 Duke Kunshan University, Suzhou, China

**Keywords:** Mitochondrial genome, Phylogenetics

## Abstract

*Gleditsia sinensis* is an endemic species widely distributed in China with high economic and medicinal value. To explore the genomic evolution and phylogenetic relationships of *G. sinensis*, the complete mitochondrial (mt) genome of *G. sinensis* was sequenced and assembled, which was firstly reported in *Gleditsia*. The mt genome was circular and 594,121 bp in length, including 37 protein-coding genes (PCGs), 19 transfer RNA (tRNA) genes and 3 ribosomal RNA (rRNA) genes. The overall base composition of the *G. sinensis* mt genome was 27.4% for A, 27.4% for T, 22.6% for G, 22.7% for C. The comparative analysis of PCGs in Fabaceae species showed that most of the ribosomal protein genes and succinate dehydrogenase genes were lost. In addition, we found that the *rps4* gene was only lost in *G. sinensis*, whereas it was retained in other Fabaceae species. The phylogenetic analysis based on shared PCGs of 24 species (22 Fabaceae and 2 Solanaceae) showed that *G. sinensis* is evolutionarily closer to *Senna* species. In general, this research will provide valuable information for the evolution of *G. sinensis* and provide insight into the phylogenetic relationships within the family Fabaceae.

## Introduction

Mitochondria are semi-autonomous organelles in eukaryotic cells, and they have relatively independent transcription and translation systems^1^. Mitochondria can provide ATP and other energy required for life activities through oxidative phosphorylation^2,3^. At present, the serial endosymbiosis theory is the most popular theory explaining the origin of mitochondria, which suggests that mitochondria originated from an endosymbiotic α-proteobacteria^4^. Most of the published complete mt genomes are from animals, protists and fungi. In contrast, the available plants mt genomes are very scarce. In 1992, the first mt genome sequencing of the land plant *Marchantia polymorpha* was completed^5^. To date, NCBI (National Center for Biotechnology Information, https://www.ncbi.nlm.nih.gov/) has collected 333 complete plant mt genomes. There is no doubt that with the development of DNA sequencing technology, the number of available plant mitochondrial sequences will increase rapidly.

The higher plants mt genomes vary dramatically in size and structure organization^6^. The length ranges from 66 kbp of *Viscum scurruloideum*^7^ to 11.3 Mbp of *Silene conica*^8^. Paradoxically, in most plants, the mitochondrial sequences evolve very slowly^9^ and the mutation rate is quite low^10^. Compared with animal mt genomes, plant mt genomes are usually large and complex^3,11^. The complexity of the plant mt genome mainly due to the presence of a large number of non-coding regions and the introgression of foreign DNA from the chloroplast or nuclear genome^12^. Despite the plant mt genome is relatively large, it contains fewer genes than its plastid counterpart, and the number of known genes is usually between 50 and 60^1,13^. The structure of the plant mt genome is usually circular, while linear form also exists in some species, such as the rice (*Oryza sativa*)^14^. The higher plants mt genome is characterized by repeat sequences^15,16^, which accounts for 2%-60% of the total genome size^17^. Some repeat sequences are species-specific and can be used as genome-specific genetic markers to study the evolutionary relationship between species^18^.

Fabaceae is the third-largest angiosperm family after Asteraceae and Orchidaceae^19^. Fabaceae plants are used in many aspects of human life, including food, wood, medicine, textiles, ornamental and horticultural plants^20^. *G. sinensis*, a kind of Fabaceae plant widely distributed throughout China^21^, provide a wide array of benefits. It plays an important role in the conservation and maintenance of soil and water resources due to its drought resistance and low requirements on the soil. In addition, *G. Sinensis* saponin is effective in decontamination, foaming, which is widely used in the production of cosmetics and detergents with high economic value^22^. The fruits and thorns of *G. Sinensis* with remarkable antioxidant, anti-tumor, antiviral, antibacterial, and anti-allergic activities ^23^, are used as medicinal herbs in China and have been used in the treatment of cancer, carbuncles, skin diseases as well as other diseases^23,24^. However, its mt genome has not been determined, which highly limits the process of molecular research on *G. sinensis*.

In this study, we assembled the complete mt genome of *G. sinensis*, which is the first mt genome for *Gleditsia*. We analyzed its gene content, repeat sequences, codon usage bias, synonymous and nonsynonymous substitution rate. Besides, gene loss and phylogenetic analyses were performed by comparisons with other Fabaceae plants mt genomes. Our data will provide valuable information for studying the evolutionary processes of the *G. sinensis* mt genome.

## Results and discussion

### Genome features

**Figure 1:**
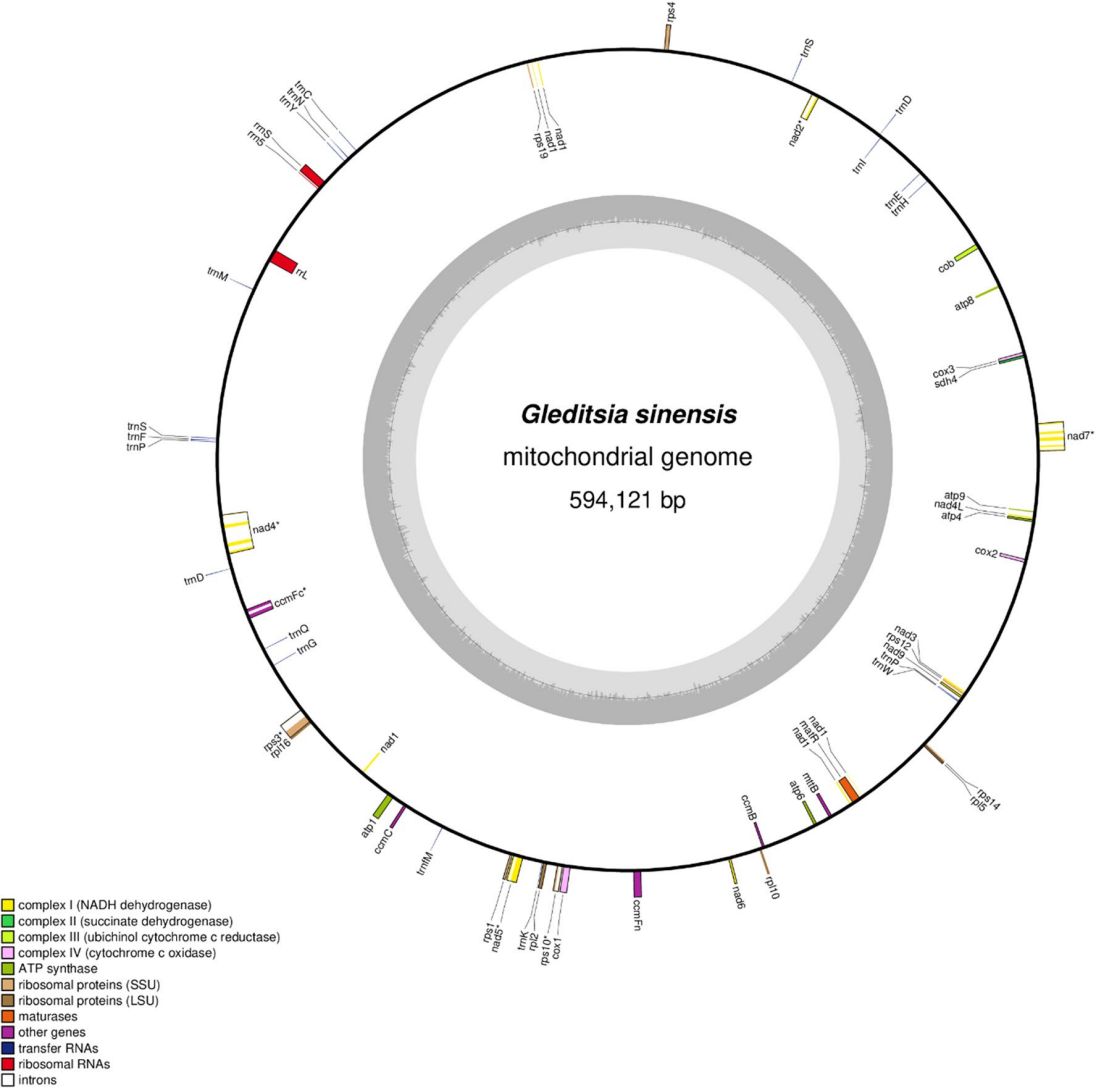
Genome map of the *G. sinensis* mt genome. Genes belonging to the functional group are color-coded on the circle as transcribed clockwise (outside) and transcribed counter-clockwise (inside). The darker gray in the inner circle represents the GC content, while the lighter gray represents the AT content.

The complete mt genome of *G. sinensis* is 594,121 bp in length with a circular structure (Fig. 1), and its size is similar to the mt genomes of some Fabaceae plants, such as *V. faba* (588,000 bp)^25^, *L. coriaria* (601,574 bp)^26^ and *T. indica* (607,282 bp)^26^. The base composition is as follows: A (27.4%), T (27.4%), C (22.7%), G (22.6%), and GC content is 45.3%. A total of 57 genes were identified in the *G. sinensis* mt genome, including 37 PCGs, 19 transfer RNA genes and 3 ribosomal RNA genes (Table 1). As shown in Table 2, the PCGs in the *G. sinensis* mt genome account for 5.11% of the entire genome with a total length of 30,336, while non-coding regions account for 90.65% of the entire genome, with a total length of 538,550. The total length of tRNA genes and rRNA genes comprise 0.24% and 0.85% of the entire mt genome, respectively. There exist 12 introns in 37 PCGs, accounting for 3.16% of the genome. Among them, *nad2, nad5, ccmFc, rps3* and *rps10* contain one intron, and *nad4* and *nad7* contain three and four introns, respectively (Table 1). Additionally, a protein-coding gene (*nad1*) and three tRNA genes (*trnP, trnD, trnS*) were found to contain two copies (Table 1).

**Table 1:** Gene annotation of the *G. sinensis* mt genome.

Category	Group	Genes
Mitochondrial respiratory chain related genes	Complex I	*nad1*(× 2)*, nad2*^a^*, nad3, nad4*^b^*, nad4L, nad5*^a^*, nad6, nad7*^b^*, nad9*
	Complex II	*sdh4*
	Complex III	*cob*
	Complex IV	*cox1, cox2, cox3*
	Complex V	*atp1, atp4, atp6, atp8, atp9*
	Cytochrome c synthesis	*ccmFn, ccmB, ccmC, ccmFc*^a^
Transcription and translation related genes	Ribosomal proteins	*rpl2, rpl10, rpl5, rpl16, rps1, rps3*^a^*,* *rps4, rps10*^a^*, rps12, rps14, rps19*
RNA genes	Transfer RNA	*trnP*(× 2)*, trnW, trnK, trnfM,* *trnG, trnQ, trnD*(× 2)*, trnF,* *trnS*(× 2)*, trnM, trnY, trnN, trnC, trnI, trnE, trnH*
	Ribosomal RNA	*rrnL, rrn5, rrnS*
Other genes	Maturase	*matR*
	Methyltransferase	*mttB*

^a^Genes with one intron, ^b^ genes with at least two introns.

**Table 2:** Genomic features of *G. sinensis* mt genome.

Feature	Size (bp)	Proportion in Genome (%)
Whole genome	594,121	100
PCGs^a^	30,336	5.11
introns^a^	18,752	3.16
tRNA genes^a^	1,420	0.24
rRNA genes^a^	5,063	0.85
Non-coding regions	538,550	90.65

^a^PCGs, introns, tRNA genes, and rRNA genes belong to coding regions.

### Codon usage analysis

**Figure 2:**
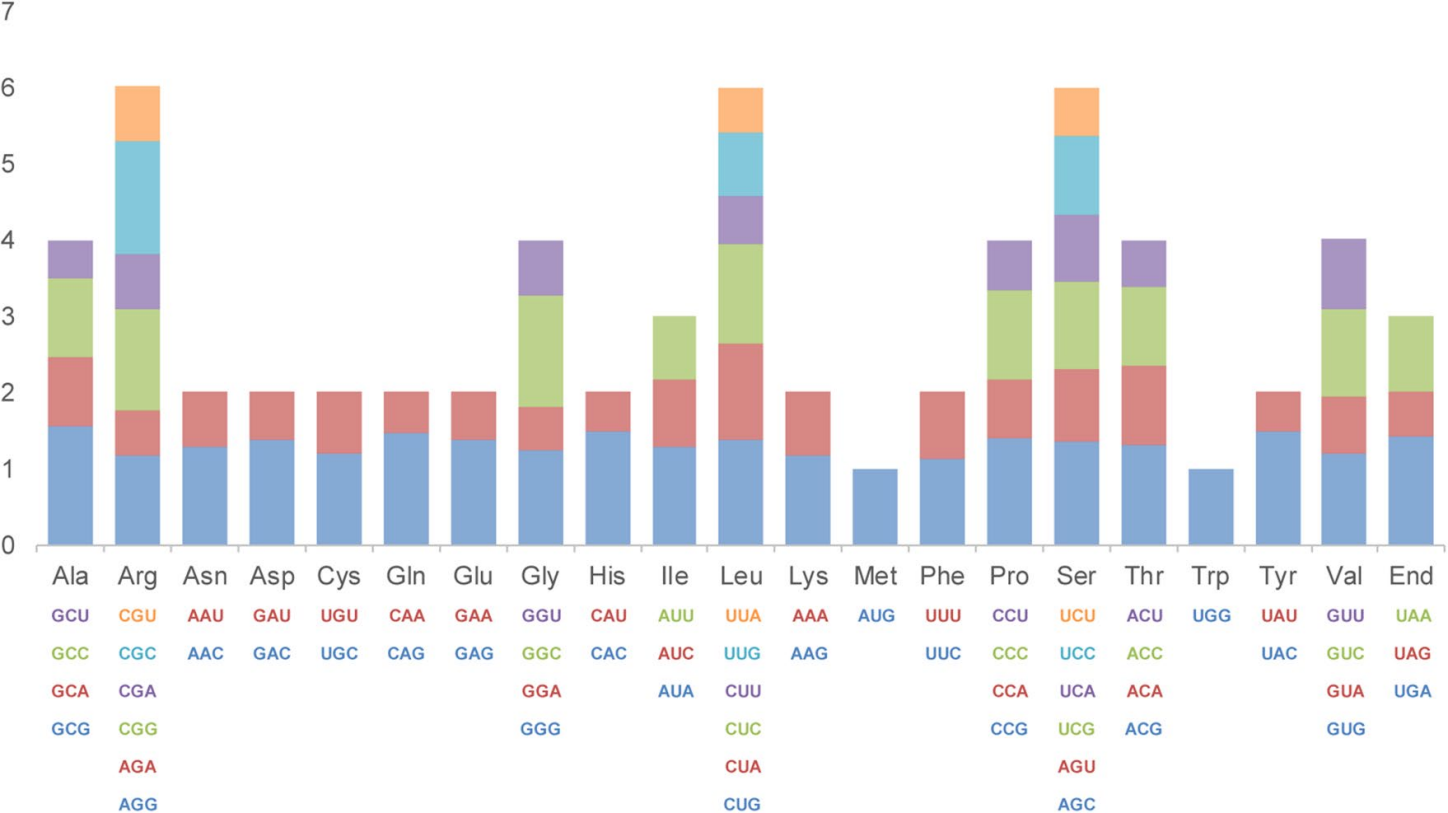
RSCU based on PCGs of the mt genome of *G. sinensis*.

Relative synonymous codon usage (RSCU) refers to the relative probability of a specific codon between the synonymous codons encoding the corresponding amino acid^27^. RSCU = 1 indicates that there is no preference for codon usage, while RSCU > 1 indicates that the codon is a used relatively frequently codon^28,29^. The 37 PCGs of the *G. sinensis* mt genome contained 10,112 codons (Supplementary Table S1). Among them, 1057 (10.45%) encoded leucine (Leu) while only 147 (1.45%) encoded cysteine (Cys), which were the most and least used amino acids in the *G. sinensis* mt genome, respectively (Table S1). The AT content of the first, second, and third codon positions was 52.01%, 56.59% and 61.26%, respectively. The high AT content at the third codon position was similar to other reported higher plants mt genomes^26,27^. Apart from UGG, all preferred synonymous codons (RSCU > 1) end in either A or U (Fig. 2).

### Repeat sequences

**Figure 3:**
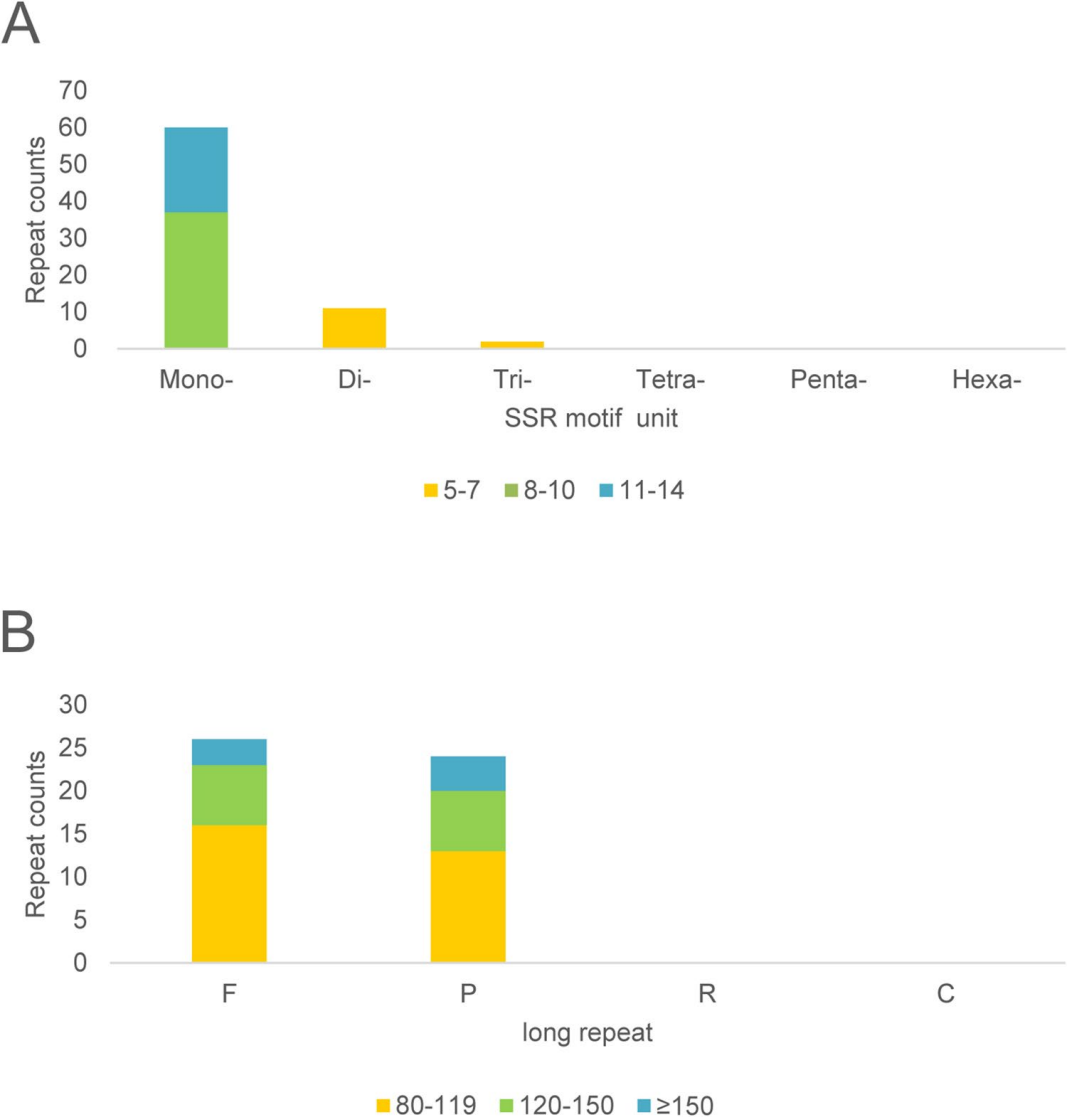
Analyses of repeats in the *G. sinensis* mt genome. (**A**) The number of different types of SSRs. (**B**) The number of different types of long repeats.

The angiosperms mt genomes are characterized by repeat sequences, which play an important role in biological evolution, genetic regulation and gene expression^32,33^. SSRs are tandem repeats with 1–6 nucleotides as the basic unit^34^, which are particularly abundant in plant genomes and have an important impact on the function and evolution of the genome^21^. SSRs are generally used as DNA markers for population genetic studies due to the advantages of high polymorphism^35^. In the present study, we identified 71 SSRs with a total length of 718 bp, including 11 dinucleotides (15.49%), two trinucleotides (2.82%), and 58 mononucleotides (84.51%), while tetranucleotides, pentanucleotides, and hexanucleotides were not identified in the mt genome (Fig. 3A). Among them, the most abundant repeat sequences were mononucleotides, which suggests that mononucleotide repeats may contribute more to genetic variation than other SSRs^36^. Further analysis of the repeat unit of SSRs showed that 80.28% of mononucleotides were A/T, while G/C only accounted for 4.23% (Table S2). The higher AT contents in mononucleotide repeats of *G. sinensis* mt genome was congruent with other reported Fabaceae plants^37^. The identification of featured SSRs in this study can provide valuable resources on developing markers for phylogenetic research and population studies of *G. sinensis.*

The sequences with a repeat unit longer than 30 bp were regarded as long repeats, including forward repeats (F), palindromic repeats (P), reverse repeats (R) and complement repeats (C). We identified 50 long repeat sequences in *G. sinensis* mt genome, ranging from 86 to 270 bp, including 26 forward repeats and 24 palindromic repeats. Most long repeats were 80–119 bp in length, and only 7 repeats were longer than 150 bp (Fig. 3). Repeat sequences, especially long repeats, have important impacts on the structure of plant mt genomes, and they are positively correlated with the size of the genome^12^.

### Synonymous and nonsynonymous substitution rate

**Figure 4:**
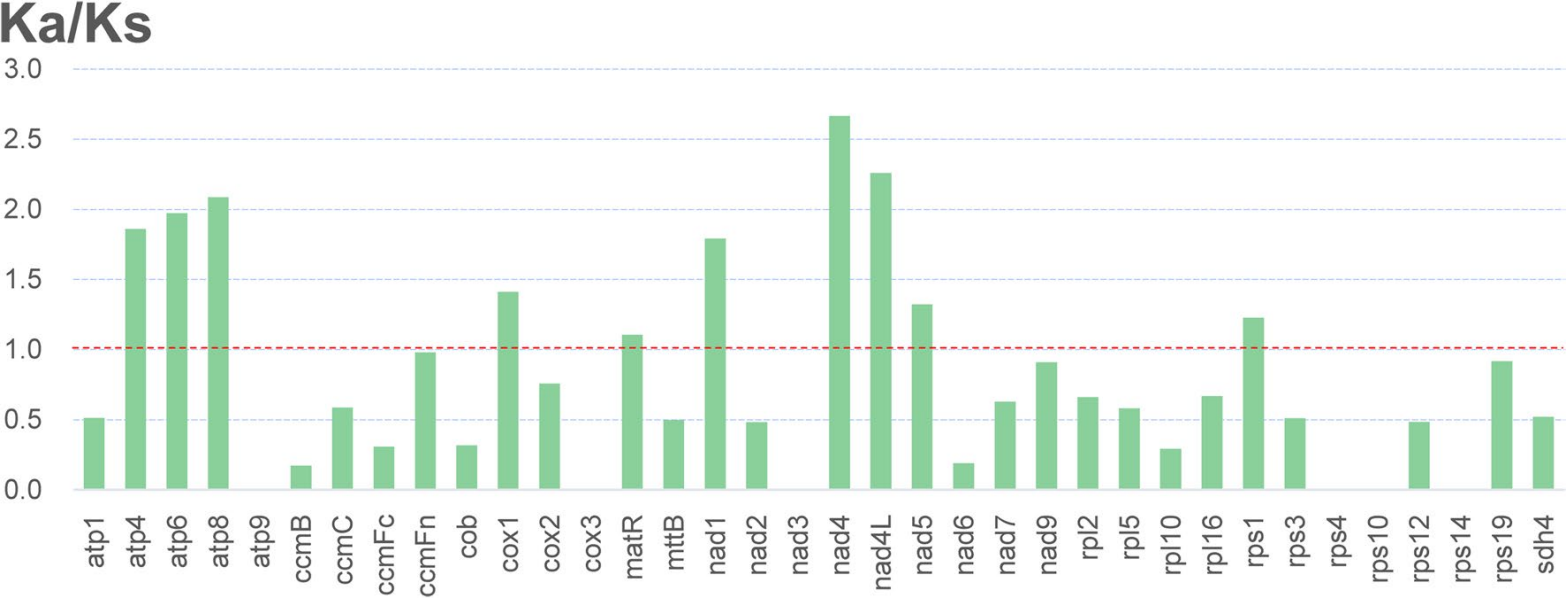
The Ka/Ks ratios for 37 PCGs of *G. sinensis.*

The calculation of Ka/Ks ratio is important for understanding the dynamics of molecular evolution^38,39^. This ratio can infer whether the PCGs are under selective pressure. Ka/Ks = 1 indicates neutral mutation, Ka/Ks < 1 indicates negative (purifying) selection, and Ka/Ks > 1 indicates positive (diversifying) selection. In this study, all of the PCGs of the *G. sinensis* mt genome were used to calculate the Ka/Ks ratios. As shown in Fig. 4, the Ka/Ks ratios of most PCGs were less than 1, indicating that most of the PCGs were under purification selection. These mitochondrial genes that experienced purification selection may play a vital role in stabilizing the normal function of mitochondria^37^. In addition, the Ka/Ks ratios of *atp4, atp6, atp8, cox1, matR, nad1, nad4, nad4L, nad5, rps1* were all greater than 1, and almost all of these genes belong to mitochondrial respiratory chain related genes category, indicating that they were under positive selection, which suggests that some advantages had emerged during evolution^37^.

### Gene loss

**Figure 5:**
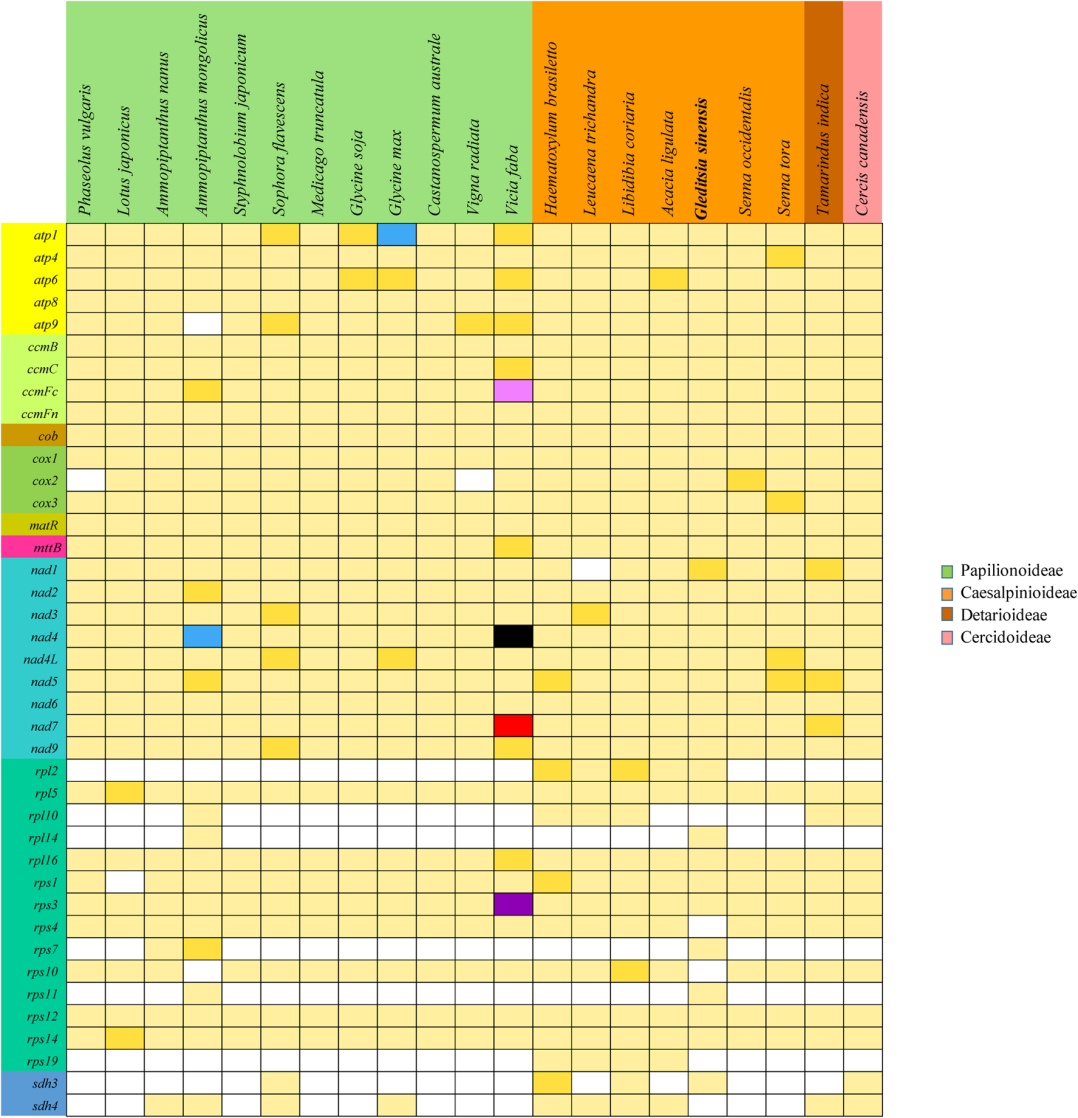
Distribution of PCGs in 22 Fabaceae plant mt genomes. White boxes indicate that the gene is not present in the mt genomes. Light yellow, golden, blue, purple, black, pink and red boxes indicate that one, two, three, four, five, six and twelve copies exist in the particular mt genomes, respectively. Light green, orange, brown and rose red boxes indicate that Papilionoideae, Caesalpinioideae, Detarioideae, Cercidoideae, respectively.

During the evolution of the angiosperm mt genome, the loss of PCGs occurred frequently^40,41^. In this study, we compared the distribution of PCGs in 22 Fabaceae plant mt genomes (Table S3). As shown in Fig. 5, most PCGs were conserved, especially for mitochondrial respiratory chain related genes, maturase and methyltransferase genes. In contrast, the ribosomal protein and succinate dehydrogenase genes were highly variable. The *rpl2*, *rpl10*, *rpl14*, *rps7*, *rps11*, *rps19*, *sdh3*, *sdh4* genes were lost in most mt genomes, which is understandable because that ribosomal protein and succinate dehydrogenase genes are frequently lost or transferred to the nucleus during the evolution of angiosperm mt genomes (e.g. *rps10*, *rpl2*, *sdh3*, *sdh4*)^26,41,42^. A total of five genes were lost in the *G. sinensis* mt genome, including four ribosomal protein genes (*rpl10, rps4, rps10, rps19*) and one succinate dehydrogenase gene (*sdh4*). The *rps10* gene was only lost in *G. sinensis* but it was retained in other Caesalpinioideae species. In addition, we found that the *rps4* gene was only lost in *G. sinensis* but it was retained in other Fabaceae species. Interestingly, this gene has not been found lost in other plant mt genomes, yet. Therefore, it is an open question as to whether *rps4* was lost for the reason that its function may no longer be needed for *G. sinensis*, or whether it was functionally transferred to the nucleus^43,44^.

### Phylogenetic analyses

**Figure 6:**
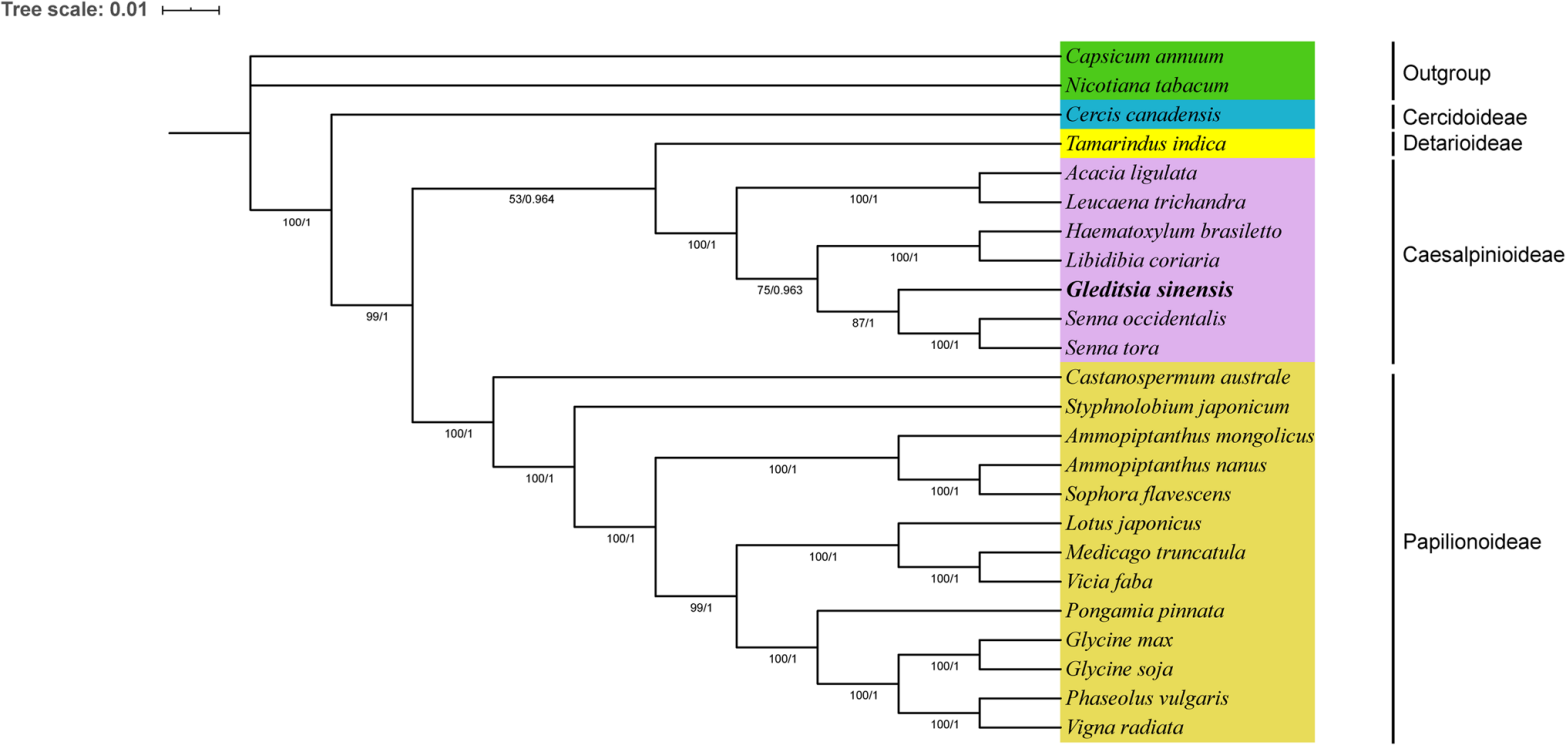
Maximum likelihood phylogenies of *G. sinensis* within Fabaceae. Relationships were inferred using 17 conserved PCGs of 24 plant mt genomes. Numbers on each node are bootstrap support values and posterior probabilities. The scale indicates the number of nucleotide substitutions per site.

The higher plant mt genomes evolve slowly, and its mutation rate is significantly low^1,8,10^, which makes it a useful tool for phylogenetic research^45^. In this study, phylogenetic analyses were performed based on the 24 plants mt genomes, including 22 Fabaceae (*P. vulgaris, H. brasiletto, L. coriaria, T. indica, S. flavescens, A. ligulate, G. soja, L. trichandra, A. mongolicus, S. japonicum, S. occidentalis, S. tora, M. truncatula, G. max, G. sinensis, L. japonicus, P. pinnata, V. radiata, C. canadensis, C. austral, A. nanus, V. faba*) and two Solanaceae *(Capsicum annuum, Nicotiana tabacum* ). Meanwhile, two Solanaceae species were used as outgroups. The ML tree and BI tree were constructed based on 17 shared PCGs (*atp6, ccmB, ccmC, ccmFn, cox1, cox3, matR, nad2, nad4, nad5, nad6, nad7, nad9, rpl16, rps3, rps4, rps12*). The ML and BI trees shared a consistent typology. As shown in Fig. 6, all Fabaceae plants were clustered within a lineage distinct from the outgroup. Most nodes in the ML and BI trees had high support values (bootstrap proportions ≥ 75, posterior probabilities ≥ 0.963), whereas the support value of the clade Detarioideae and Caesalpinioideae was only 53 in the ML tree. The phylogenetic relationship of the four subfamilies was described as (Cercidoideae + (Papilionoideae + (Detarioideae + Caesalpinioideae))). The tree strongly support the separation of Cercidoideae from the clade (Papilionoideae, Detarioideae, Caesalpinioideae), with bootstrap proportions = 100, posterior probabilities = 1, which was consistent with a previous study report^26^. It was worth noting that the *G. sinensis* and two *Senna* species were clustered into one clade with a bootstrap support value of 87 and a posterior probability of 1, which indicates that *G. sinensis* were evolutionarily closer to *Senna* species within the Fabaceae family. The phylogenetic tree constructed in this study could not reflect the true phylogenetic relationship of Fabaceae for the fact that few Fabaceae mt genomes have been sequenced. To illustrate more accurately the evolutionary relationship among Fabaceae species, it is necessary to use more species to analyze the phylogeny.

## Methods

### DNA extraction and sequencing

The fresh leaves of *Gleditsia sinensis* used in this study were collected from the Chinese Medicine Botanical Garden of Tianjin University of Traditional Chinese Medicine (117.06°E, 38.96°N), and it was identified by Prof. Tianxiang Li. The collection of *Gleditsia Sinensis* was approved by Tianjin University of Traditional Chinese Medicine and was conducted in accordance with the standards of "Medicine Mountain Collection of Tianjin University of Traditional Chinese Medicine". The voucher specimens were deposited in the State Key Laboratory of Component-Based Chinese Medicine, voucher No.G20191120. The collected leaves were quickly frozen in liquid nitrogen and then stored at − 80 °C until DNA extraction. Total genomic DNA was extracted by using the extract Plant DNA kit (QIAGEN, Germany). Truseq Nano DNA HT sample preparation kit (Illumina USA) was used to construct a 350 bp insert-sized DNA sequencing library, which was later sequenced with a paired-end read length of 2 × 150 bp on Illumina HiSeq X Ten platform following the standard Illumina protocols (Illumina, San Diego, CA).

### Mitochondrial genome assembly and annotation

A total of 14,836,699 raw reads of *G. sinensis* were produced by Illumina pair-end sequencing, and 14,794,823 clean reads were retained after the quality checking by FastQC. The base quality value Q20 and Q30 were 94.16% and 86.35%, respectively. Subsequent analyses were based on the filtered high-quality sequences. For mt genome assembly, high-quality DNA sequencing reads were mapped to reference mt genome of *Senna occidentalis* (NCBI accession number NC_038221) using Geneious^46^ to get the sequence of *cox1*, the number of iterations was set to 5 times. Then, the *G. sinensis* mitochondrial genome was de novo assembled using NOVOPlasty3.7.2^47^, with *cox1* sequence set as seed and K-mer length of 39. The N50 and N90 of the obtained contigs were 64428 bp and 18438 bp, respectively. In order to obtain a high-quality mt genome, the base of the genome was corrected based on high-quality DNA sequencing data by using BWA software^48–50^, and a total of 96 bases were corrected. Finally, to determine whether the assembled contig is a circular structure, we designed primers based on the base sequence at the head and tail of the contig and performed PCR amplification (Table S4). The results confirmed that the *G. sinensis* mt genome was a typical circular molecule (Figure S1).

The mt genome was annotated using MITOFY^12^ (http://dogma.ccbb.utexas.edu/mitofy/) and GeSeq^51^ (https://chlorobox.mpimp-golm.mpg.de/geseq.html) and was manually checked and adjusted the annotation using *Senna occidentalis* as the reference sequence. The online tRNAscan-SE search server (http://lowelab.ucsc.edu/tRNAscan-SE) was used to annotate the tRNA gene to determine its position, and the parameter settings were default. The start and stop codons of protein-coding genes were manually adjusted to fit open reading frames. The mt genome of *G. sinensis* was visualized using OGDRAW^52^. The mt genome of *G. sinensis* was deposited in NCBI GenBank under accession number MT921986.

### Codon usage and substitution rate calculation

The relative synonymous codon usage (RSCU) was calculated by MEGA X^53^. The Ka/Ks ratios were calculated individually on each protein-coding gene of *G. sinensis* by DnaSP v6^54^, and *Acacia ligulata* (NCBI accession number NC_040998) was used as an outgroup.

### Repeat sequence

The position and type of SSR (Simple Repeated Sequence) were detected using the microsatellite identification tool MISA-web^55^ (https://webblast.ipk-gatersleben.de/misa/) with parameters set to 10, 5, 4, 3, 3 and 3 for mono-, di-, tri- tetra-, penta-, and hexanucleotides, respectively. The size and position of long repeat sequences, including forward, palindromic, reverse and complement repeats, were detected by REPuter^56^ (http://bibiserv.tech--fak.uni-bielefeld.de/reputer/), with a minimal repeat size of 30, and a hamming distance of 3.

### Phylogenetic analyses

To better infer the phylogenetic relationship within the Fabaceae family, 24species (22 Fabaceae species and 2 outgroups) were selected to construct a phylogenetic tree. We extracted the nucleotide sequences of shared PCGs from these mt genomes. The 17 shared PCGs were aligned individually using PhyloSuite v1.2.1^57^, and the alignment was manually adjusted. All aligned PCGs were then concatenated. Maximum likelihood (ML) analysis was performed using IQ-TREE^58^ under the model automatically selected. The Bayesian inference (BI) was implemented with MrBayes 3.2.6^59^ under JC + I + G model determined from the ModelFinder^60^.

## Supplementary Information

Supplementary Information.

## Data Availability

The genome sequence data that support the findings of this study are openly available in GenBank of NCBI at (https://www.ncbi.nlm.nih.gov/) under the accession no.MT921986. The associated BioProject, SRA, and Bio-Sample numbers are PRJNA726335, SRR14368777 and SAMN18927823 respectively.
