# Supplementary material for: Insights into molecular structure, genome evolution and phylogenetic implication through mitochondrial genome sequence of Gleditsia sinensis
Source: Sci Rep. 2021 Jul 21;11:14850. doi: 10.1038/s41598-021-93480-6 (PMC8295344; doi:10.1038/s41598-021-93480-6)
Supplement: Supplementary file 1 — Supplementary Information. [file 41598_2021_93480_MOESM1_ESM.pdf]

Supporting Information

Title: Insights into molecular structure, genome evolution and phylogenetic implication through mitochondrial genome sequence of *Gleditsia sinensis*

Hongxia Yang[1,3], Wenhui Li[1,3], Xiaolei Yu[1,3], Xiaoying Zhang[1], Zhongyi Zhang[2], Yuxia Liu[1], Wenxiu Wang[1], Xiaoxuan Tian [1,*]

1 State Key Laboratory of Component-Based Chinese Medicine, Tianjin University of Traditional Chinese Medicine, Tianjin, 300193, China.
2 Duke Kunshan University.
*Corresponding: tian_xiaoxuan@tjutcm.edu.cn
3 These authors contributed equally to this work

Table S1. Relative synonymous codon usage (RSCU)

Codon	Count	RSCU	Codon	Count	RSCU	Codon	Count	RSCU	Codon	Count	RSCU
UUU(F)	364	1.14	UCU(S)	211	1.36	UAU(Y)	224	1.5	UGU(C)	89	1.21
UUC(F)	277	0.86	UCC(S)	148	0.95	UAC(Y)	75	0.5	UGC(C)	58	0.79
UUA(L)	244	1.39	UCA(S)	175	1.13	UAA(*)	17	1.42	UGA(*)	12	1
UUG(L)	220	1.25	UCG(S)	138	0.89	UAG(*)	7	0.58	UGG(W)	153	1
CUU(L)	230	1.31	CCU(P)	206	1.4	CAU(H)	182	1.49	CGU(R)	141	1.17
CUC(L)	110	0.62	CCC(P)	114	0.77	CAC(H)	62	0.51	CGC(R)	72	0.6
CUA(L)	150	0.85	CCA(P)	172	1.17	CAA(Q)	203	1.48	CGA(R)	160	1.33
CUG(L)	103	0.58	CCG(P)	98	0.66	CAG(Q)	71	0.52	CGG(R)	86	0.71
AUU(I)	324	1.28	ACU(T)	169	1.31	AAU(N)	213	1.29	AGU(S)	160	1.03
AUC(I)	224	0.88	ACC(T)	134	1.04	AAC(N)	117	0.71	AGC(S)	100	0.64
AUA(I)	214	0.84	ACA(T)	134	1.04	AAA(K)	266	1.17	AGA(R)	178	1.48
AUG(M)	260	1	ACG(T)	78	0.61	AAG(K)	189	0.83	AGG(R)	87	0.72
GUU(V)	189	1.2	GCU(A)	243	1.56	GAU(D)	221	1.38	GGU(G)	217	1.25
GUC(V)	118	0.75	GCC(A)	139	0.89	GAC(D)	100	0.62	GGC(G)	96	0.55
GUA(V)	182	1.15	GCA(A)	161	1.04	GAA(E)	289	1.37	GGA(G)	255	1.47
GUG(V)	143	0.91	GCG(A)	79	0.51	GAG(E)	134	0.63	GGG(G)	127	0.73

Table S2. Number of different SSR repeat units

Repeats	5	6	7	8	9	10	11	12	13	14	total
A/T	-	-	-	-	-	36	12	6	1	2	57
C/G	-	-	-	-	-	1	1	-	1	-	3
AC/GT	-	-	1	-	-	-	-	-	-	-	1
AG/CT	-	2	-	-	-	-	-	-	-	-	2
AT/AT	-	6	2	-	-	-	-	-	-	-	8
AAT/ATT	2	-	-	-	-	-	-	-	-	-	2

Table S3. NCBI accession number.

**NCBI accession number**	**species**
NC_045135	*Phaseolus vulgaris*
NC_045040	*Haematoxylum brasiletto*
NC_045039	*Libidibia coriaria*
NC_045038	*Tamarindus indica*
NC_043897	*Sophora flavescens*
NC_040998	*Acacia ligulata*
NC_039768	*Glycine soja*
NC_039738	*Leucaena trichandra*
NC_039660	*Ammopiptanthus mongolicus*
NC_039596	*Styphnolobium japonicum*
NC_038221	*Senna occidentalis*
NC_038053	*Senna tora*

NC_029641	*Medicago truncatula*
NC_020445	*Glycine max*
NC_016743	*Lotus japonicus*
NC_016742	*Pongamia pinnata*
NC_015121	*Vigna radiata*
MN017226	*Cercis canadensis*
MK426679	*Castanospermum australe*
MH127920	*Ammopiptanthus nanus*
KC189947	*Vicia faba*
NC_024624	*Capsicum annuum*

Table S4. The name and sequence of the primer and the sequence of the amplicon.

Primer name	Sequence (5' to 3')	Amplicon sequence
zjF	GCTGCTACCGAATGAAGT	GCTGCTACCGAATGAAGTTCGTCCTTCCCCCCCCCCCCCGAA<br>CCCTATAATAAAGTGCGGGCCCCCGTAGATAGATGCCCCATAT<br>AGCCCCTCCTCTTCCGGGGTTGGGCCGAAAAAAGAATCGAAT<br>TGGGTTGAGAGTGAAAATGAAAAAGAAAAATCGTATTATTAA<br>TTATAATAATACCAAAAATATGAAAAGCAAGTGCCTTAGTTCTT
zjR	AGCAAGTGCCTTAGTTCTT	

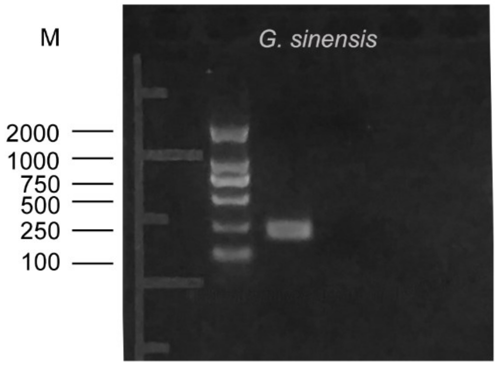

Figure S1. PCR amplification result. The left bands are DL2000 DNA marker, and the right band is the PCR product of *G. sinensis*. The length of the PCR product is consistent with the expected result. The molecular-weight size marker on the side of the gel is expressed in bp. This figure shows the full-length gel.
